# Perceptions and use intentions of flavored versus unflavored tobacco products among young adults in Georgia: A cross-sectional study

**DOI:** 10.18332/tpc/208691

**Published:** 2025-10-06

**Authors:** Tamar Abuladze, Carla Berg, George Bakhturidze, Lela Sturua

**Affiliations:** 1 School of Health Sciences, University of Georgia, Tbilisi, Georgia; 2 National Center for Disease Control and Public Health, Tbilisi, Georgia; 3 Milken Institute School of Public Health, The George Washington University, Washington, United States; 4 GW Cancer Center, The George Washington University, Washington, United States; 5 School of Public Health, Georgia State University, Georgia, United States; 6 Petre Shotadze Tbilisi Medical Academy, Tbilisi, Georgia

**Keywords:** young adults, e-cigarettes, harm perception, addictiveness, flavored tobacco products, social acceptability

## Abstract

**INTRODUCTION:**

Tobacco product marketing uses flavors to influence consumer perceptions, especially among youth and young adults. In Georgia, where tobacco use is among the highest in the WHO European Region, flavored products are widely available and unregulated. Limited data exist on young adults’ perceptions of flavored versus unflavored products.

**METHODS:**

This study aimed to assess how young adults in Georgia perceive flavored versus unflavored tobacco products in terms of harm, addictiveness, and social acceptability, and how these perceptions relate to their use intentions. A cross-sectional online survey (April–June 2024) included 400 participants aged 18–25 years measured perceptions on a 1–7 scale for flavored and unflavored cigarettes, e-cigarettes, and heated tobacco products. Perceptions were categorized as less, equal, or more harmful/addictive/acceptable. Multivariable logistic regression examined associations between these perceptions and past-month use and use intentions.

**RESULTS:**

Most participants perceived flavored and unflavored products similarly, but perceptions varied across product types. Flavored e-cigarettes were most often rated as more acceptable (13%) and addictive (12.5%) compared to unflavored e-cigarettes – more frequently than flavored cigarettes (9.5% acceptable, 7.5% addictive) or flavored HTPs (7% acceptable, 9.8% addictive) when compared to their unflavored counterparts. Perceiving flavored products as more harmful to self was associated with lower past-month cigarette use (adjusted odds ratio, AOR=0.18; 95% CI: 0.08–0.40), while perceiving flavored products as more addictive was associated with higher e-cigarette use (AOR=2.78; 95% CI: 1.06–7.28). Among non-past-month users, higher perceived harm to others was associated with lower intention to use flavored cigarettes (AOR=0.040; 95% CI: 0.003–0.622).

**CONCLUSIONS:**

Although most participants perceived flavored and unflavored products similarly, small differences in some perceptions contributed to differences in certain use behaviors, supporting stronger regulation of flavored products in Georgia. However, as this is a cross-sectional study, further prospective research is needed to confirm these findings and guide policy.

## INTRODUCTION

Excessive tobacco consumption is an important public health policy issue in Georgia which has one of the highest rates of smoking in the WHO European region – 28.2% of the adult population report current smoking (49.5% men and 8.4% women)^1^ and 11400 people die annually from tobacco-related diseases in the country^2^. National surveys for adults usually assess general tobacco use without distinguishing specific product types, resulting in limited data on novel tobacco products^1^; however, studies conducted among school students suggest that conventional cigarettes and electronic cigarettes (e-cigarettes) may be the most commonly used tobacco products among youth in Georgia^3,4^. Heated tobacco products (HTPs) officially entered the Georgian market in 2020^5^ and may now also be widely used among youth and young adults. According to the GYTS 2023 survey, 11.3% of Georgian students aged 13–15 years reported current use of e-cigarettes, and 10.6% had ever used HTPs^6^. These trends likely are reflected in the young adult population.

The availability of a wide range of flavors contained in novel products may be a key factor in these usage rates, as studies from various countries show that the use of flavors is a well-known tobacco industry strategy to increase product attractiveness among young people and those without a history of smoking, reduce the harshness of smoking tobacco products, and mislead individuals to believe that the products are less harmful^7,8^.

In Georgia, flavored novel tobacco products are widely available in retail settings. However, little research has assessed how young adults in Georgia perceive flavored versus unflavored tobacco products. Relatedly, there are no specific regulations or targeted public health campaigns regarding flavorings and additives. The absence of regulations and lack of communication campaigns may contribute to misperceptions about the safety and addictiveness of these products among youth or young adults. Recently, efforts to strengthen tobacco control in Georgia have progressed, in particular since 2017 when the ‘new generation’ tobacco control legislation was adopted by the Parliament of Georgia^9^. However, certain gaps remain, especially concerning flavored tobacco products and nicotine-free e-cigarettes. Current legislation in Georgia does not include explicit restrictions on flavorings or limits the number of additives used in tobacco products^9,10^. Legislation only sets a limit for the amount of concentration of tar, nicotine and carbon dioxide in filtered/unfiltered cigarettes and concentration of nicotine in e-cigarette liquid^10^, but there is no information, if any, verification of compliance with this requirement by tobacco manufacturers and importers have been. Furthermore, under current Georgian legislation, nicotine-free e-cigarettes are not classified as tobacco products, exempting them from content disclosure requirements and other tobacco control measures^9^.

Given the nuances of tobacco use and regulation in Georgia, understanding how young adults’ tobacco-related perceptions and behaviors are influenced is critical to local policy debates regarding flavored tobacco products. Thus, this study aims to examine how flavoring (flavored vs unflavored) influences young adults’ perceptions of harm, addictiveness, social acceptability, and their intention to use tobacco products in Georgia. We hypothesized that flavored products would be perceived as less harmful and addictive and more socially acceptable than unflavored products, and that these perceptions would be associated with greater use intentions.

## METHODS

### Study design and setting

This cross-sectional study analyzed survey data to investigate the role of flavoring (flavored vs unflavored) in perceptions of harm, social acceptability, addictiveness and future intent to use tobacco products among young adults in Georgia. The survey was conducted from April to June 2024, using a structured, self-administered online questionnaire hosted on the SurveyMonkey platform and disseminated via targeted advertisements on Facebook and Instagram. The online setting allowed for broad geographical coverage across urban and rural areas and was particularly appropriate for the target population of young adults aged 18–25 years, who are among the most active users of digital and social media platforms in Georgia. Ads posted on Facebook and Instagram said ‘If you are Georgian and aged between 18 and 25 years, please give 15 minutes for this questionnaire on tobacco perception’. Eligibility was confirmed through screening questions at the beginning of the online survey. Only participants who reported being aged 18–25 years and currently residing in Georgia were able to proceed to the full questionnaire. Once potential participants clicked on ads they were sent to a study webpage which provided a study description, the informed consent form, and eligibility screening form. Individuals who were eligible and consented were then sent to the online survey.

### Participants

Eligible participants were residents of Georgia aged 18–25 years, which was assessed through self-report during the eligibility screening stage. Those who did not meet these criteria (e.g. aged <18 years, aged >25 years, or residing outside Georgia) were screened out automatically.

The required sample size was calculated using Cochran’s formula, resulting in a final sample size of 384 respondents (p=0.5). To reach the target sample size, 1186 people opened the questionnaire link. Of these, 1102 individuals began the survey, and 825 completed eligibility screening and started filling out the questionnaire. Of the 825 who passed eligibility screening and began the questionnaire, 400 completed all relevant questions and met inclusion criteria for the final analysis. Data collection was concluded once a total of 400 participants had successfully completed the full survey. To reduce duplicate entries, the online platform restricted participation to one response per IP address.

### Measures and variables

The survey included structured questions to assess participants’ tobacco use behaviors, perceptions of flavored and unflavored tobacco products, future use intentions, and sociodemographic characteristics. The questionnaire was designed so that certain questions appeared based on the participant’s previous answers to ensure that participants only saw questions relevant to their experience. The questionnaire was developed by the research team based on prior literature on tobacco product perceptions, including items adapted from existing international surveys (e.g. GYTS, GATS). The final instrument was reviewed for relevance and clarity by tobacco control experts in Georgia.

### Tobacco product use behaviors

Before starting questions on tobacco products, brief product descriptions with a corresponding photo and reference to the most well-known brand names available on the Georgian market, were provided to ensure participants understood the terms. Participants were asked whether they had ever tried three types of tobacco products: cigarettes, e-cigarettes, and HTPs. Participants who reported lifetime use of each product were then asked to report the number of days they had used that product in the past 30 days. Participants were classified as past-month use or no past-month use of each product type.

Among those with past-month use, for each product used in the past 30 days, follow-up questions assessed whether the product they typically used was tobacco-flavored or flavored otherwise (e.g. mint, menthol, fruit), with an additional option for ‘don’t know/don’t remember’. Those who indicated using flavored products were categorized as flavored product use, while those who reported using tobacco-flavored products or selected ‘don’t know/don’t remember’ were categorized as unflavored product use for analysis.

### Perceptions of harm, addictiveness, and social acceptability

Participants were asked to rate their perceptions of flavored and unflavored versions of each product (i.e. cigarettes, e-cigarettes, HTPs) by asking: ‘How [addictive, harmful to one’s health, harmful to others, socially acceptable] do you think the following products are?’. Each perception was assessed using a 7-point Likert scale (1= ‘not at all’ to 7= ‘extremely’).

Composite scores were calculated for each perception domain by averaging responses across product types. For example, perceived harm to self of flavored products was computed as the average rating across flavored cigarettes, e-cigarettes, and HTPs. The same approach was used for unflavored products. Based on these composite scores, participants were categorized as perceiving flavored products as ‘less than’, ‘equally’, or ‘more than’ unflavored products on each dimension.

### Future use intentions

All participants were asked how likely they were to use flavored and unflavored versions of each product type in the next 12 months, using a 7-point Likert scale. These ratings were used to calculate mean use intention scores. The original 7-point scale was recoded into a binary variable: responses from 1 to 4 were categorized as low intention, and responses from 5 to 7 as high intention. This binary variable was used as the outcome in the logistic regression analyses. In additional analyses, use intentions among those reporting no past-month use were compared across the three perception categories (less than, equally, or more than unflavored products) for each perception domain – harm, addictiveness, and social acceptability – to examine how these perceptions influenced the likelihood of future use.

For the purposes of analysis, perception variables (perceived harm, addictiveness, and social acceptability) were treated as exposures, and future intention to use flavored tobacco products was treated as the primary outcome variable in the regression models.

### Sociodemographic variables

Demographic characteristics included sex, education level, employment status, and annual income. Age was not assessed, as all participants were within the eligible range of 18 to 25 years. Participants were asked to report their sex assigned at birth (male or female), highest level of education (seven options), main employment status over the past 12 months (ten options), and annual household income (four categories). For analysis, responses were recoded into the following categories to ensure adequate group sizes: sex was used as reported (male, female); education level was grouped as ‘no higher education’ (less than university degree) and ‘higher education’ (Bachelor’s degree or higher); employment status was grouped as employed (government, non-government, or self-employed), student, and unemployed (including housekeeper, retired, and unemployed); income was collapsed into two groups: <15000 GEL versus ≥15000 GEL. Responses of ‘prefer not to answer’ were treated as missing. All sociodemographic variables were included as covariates and treated as potential confounders in the multivariable regression models.

### Statistical analysis

All data were analyzed using IBM SPSS Statistics, Version 23. Descriptive statistics were used to summarize participant characteristics, tobacco use behaviors, perceptions of flavored versus unflavored tobacco products, and future use intentions. Frequencies and percentages are reported for categorical variables, and means with standard deviations for continuous variables.

Bivariate analysis characterized participants’ perceptions of flavored products versus unflavored products across four domains (harm to self, harm to others, addictiveness, and social acceptability) in relation to sociodemographic covariates (sex, education level, employment status, income) and tobacco use status (past-month use of cigarettes, e-cigarettes, and HTPs) using chi-squared tests. Additionally, one-way ANOVA tests were conducted to compare mean intention-to-use scores across perception categories among participants reporting no past-month use, for each tobacco product and flavor type.

In multivariable logistic regression models, the perception variables were the primary independent variables (treated as categorical variables with ‘equally’ as the reference group), and the primary dependent variables were: past-month use of cigarettes, e-cigarettes, and HTPs; flavored product use among those with past-month use; and higher future intention to use flavored products among those reporting no past-month use. Each regression model controlled for sex and education level, but did not include employment status and income due to collinearity with education level. Adjusted odds ratios (AORs), 95% confidence intervals (CIs), and p-values were reported for all models. A p<0.05 was considered statistically significant. All statistical tests were two-sided.

Participants with missing values on key sociodemographic or outcome variables were excluded from the regression analyses using listwise deletion. A total of 16 cases (4%) were excluded due to incomplete responses or ‘prefer not to answer’ selections.

### Ethical approval

This study was approved by the Council on Ethical Issues in Biomedical Research at the University of Georgia, Tbilisi (granted 3 April 2024). Before starting the questionnaire, all participants were required to review an informed consent statement and select ‘I agree’ in order to proceed. Eligibility criteria (aged 18–25 years, residence in Georgia) were assessed immediately after consent, and only eligible participants were allowed to continue.

No personally identifying information was collected. The only technical identifier recorded was IP address, which was used solely to prevent duplicate responses and was not linked to survey content. All data were handled anonymously and stored securely for academic research purposes only.

### AI use statement

No artificial intelligence (AI) tools were used for the design, data collection, coding, or statistical analysis of this study. All analyses were conducted manually using IBM SPSS Statistics Version 23. ChatGPT-4 (OpenAI) was used to support the manuscript’s preparation, including assistance with academic phrasing, language editing, and formatting. No AI-generated content was used in interpreting findings, constructing variables, or producing tables or figures.

## RESULTS

Table 1 presents participant characteristics (N=400). The majority of the participants aged 18–25 years were female (73.0%), and most reported a higher education degree (53.8%). More than half were students (54.3%), 34% were employed, and 8.5% were unemployed. As for annual income, 77.0% reported earning <15000 GEL.

**Table 1:** Participant characteristics, cross-sectional survey, Georgia, April–June 2024 (N=400)

*Characteristics*	*n (%)*
**Sex**	
Male	108 (27.0)
Female	292 (73.0)
**Education level**	
No higher education	179 (44.8)
Higher education	215 (53.8)
Prefer not to answer	6 (1.5)
**Employment status**	
Employed	136 (34.0)
Student	217 (54.3)
Unemployed	34 (8.5)
Prefer not to answer	13 (3.3)
**Annual income** (GEL)	
<15000	308 (77.0)
≥15000	92 (23.0)
**Past-month tobacco product use**	
Cigarettes	166 (41.5)
E-cigarettes	100 (25.0)
HTPs	60 (15.0)
**Past-month flavored product use**	
Flavored cigarettes	38 (9.5)
Flavored e-cigarettes	91 (22.8)
Flavored HTPs	29 (7.2)
Any past-month tobacco use	199 (49.8)

GEL: 1000 Georgian Lari about US$370.

In terms of tobacco product use, 41.5% reported past-month cigarette use, 25.0% e-cigarette use, and 15.0% HTP use. Among those reporting past-month use, 9.5% used flavored cigarettes, 22.8% flavored e-cigarettes, and 7.2% flavored HTPs. Among the total sample, 49.8% reported past-month use of at least one tobacco product.

Table 2 presents participants’ perceptions of flavored versus unflavored tobacco products across the four dimensions and future intentions to use each product. Most participants perceived flavored (vs unflavored) products as equally addictive (71.0%) or less addictive (15.8%), while only 13.3% perceived them as more addictive. A similar picture was revealed for perceived harm to self, with 67.8% rating flavored products as equally harmful and 14.8% as less harmful, while 17.5% rated them as more harmful. Perceived harm to others showed the lowest proportion of ‘more than’ responses (6.3%), and the majority (80.3%) considered flavored and unflavored products equally harmful to others. Regarding social acceptability, 17.0% perceived flavored (vs unflavored) products as more acceptable, 73.3% equally acceptable, and 9.8% less acceptable.

**Table 2:** Perceptions of flavored tobacco products compared to unflavored products across the four dimensions of addictiveness, harmfulness (to self and to others), acceptability, and their future intentions to use each product, cross-sectional survey, Georgia, April–June 2024 (N=400)

	*Less than n (%)*	*Equally n (%)*	*More than n (%)*
**Addictiveness**			
Addictive – composite	63 (15.8)	284 (71.0)	53 (13.3)
Cigarettes	64 (16.0)	306 (76.5)	30 (7.5)
E-cigarettes	22 (5.5)	328 (82.0)	50 (12.5)
HTPs	17 (4.3)	344 (86.0)	39 (9.8)
**Harmfulness to self**			
Harmful to self – composite	59 (14.8)	271 (67.8)	70 (17.5)
Cigarettes	46 (11.5)	296 (74)	58 (14.5)
E-cigarettes	29 (7.2)	341 (85.3)	30 (7.5)
HTPs	14 (3.5)	355 (88.8)	31 (7.8)
**Harmfulness to others**			
Harmful to others – composite	54 (13.5)	321 (80.3)	25 (6.3)
Cigarettes	43 (10.8)	344 (86)	13 (3.3)
E-cigarettes	16 (4)	366 (91.5)	18 (4.5)
HTPs	12 (3)	373 (93.3)	15 (3.8)
**Acceptability**			
Acceptable – composite	39 (9.8)	293 (73.3)	68 (17.0)
Cigarettes	40 (10)	322 (80.5)	38 (9.5)
E-cigarettes	16 (4)	332 (83)	52 (13)
HTPs	20 (5)	352 (88)	28 (7)
**Intentions**			
Intention to use – composite	86 (21.5)	223 (55.8)	91 (22.8)
Cigarettes	94 (23.5)	273 (68.3)	33 (8.3)
E-cigarettes	12 (3)	285 (71.3)	103 (25.8)
HTPs	20 (5)	362 (90.5)	18 (4.5)

Across product types, flavored e-cigarettes were perceived more favorably than flavored cigarettes and HTPs. Specifically, 13.0% of participants rated flavored e-cigarettes as more socially acceptable, compared to 9.5% for flavored cigarettes and 7.0% for flavored HTPs. Similarly, 12.5% rated flavored e-cigarettes as more addictive, compared to 7.5% for flavored cigarettes and 9.8% for HTPs. In terms of perceived harm to self, 7.2% believes that flavored e-cigarettes are less harmful, compared to 11.5% for flavored cigarettes and 3.5% for flavored HTPs.

Future intention to use flavored products was generally low, with only 22.8% reporting high likelihood of using flavored products in the next 12 months. Product-specific responses indicated greater likelihood of future use for flavored e-cigarettes (25.8%) compared to flavored cigarettes (8.3%) or flavored HTPs (4.5%).

### Associations between participant characteristics and perceptions of flavored versus unflavored tobacco products

Supplementary file Tables 1a and 1b present bivariate results regarding associations between participants’ sociodemographic characteristics and perceptions of flavored versus unflavored tobacco products, showing no significant associations. Supplementary file Tables 2a and 2b present bivariate results regarding associations between participants’ use behaviors and intentions and perceptions of flavored versus unflavored tobacco products, as well as use intentions. Participants who reported past-month use of any tobacco product (p=0.022) were more likely to perceive flavored products as more harmful to others than non-users. Similarly, participants who reported past-month use of any tobacco products (p<0.001) were more likely to perceive flavored tobacco products as more harmful to self than non-users. Participants who perceived flavored products as more harmful to others reported higher intention to use unflavored cigarettes (p=0.045), as well as flavored HTPs (p=0.015) suggesting that multiple factors may influence product-specific intentions beyond perceived harm alone. Similarly, participants who perceived flavored products as more harmful to others reported higher intention to use unflavored e-cigarettes (p<0.001) and unflavored HTPs (p=0.004). No significant associations were found between perception of social acceptability and intention to use any tobacco product.

### Findings from multivariable logistic regression analyses

In multivariable logistic regression models exploring perceptions in relation to past-month tobacco use (Table 3), results showed that participants perceiving flavored products as more harmful to themselves were less likely to report past-month cigarettes use (AOR=0.18; 95% CI: 0.08–0.40, p<0.001). Also, those perceiving flavored products as less harmful to themselves had lower odds of past-month cigarette use (AOR=0.30; 95% CI: 0.16–0.54, p<0.001) and e-cigarette use (AOR=0.46; 95% CI: 0.25–0.85, p=0.013).

**Table 3:** Multiple logistic regression for predictors of past-month product use (Set A), Georgia, April–June 2024

*Predictors*	*Cigarettes*				*E-cigarettes*				*HTPs*			
	*AOR*	*95% CI*		*p*	*AOR*	*95% CI*		*p*	*AOR*	*95% CI*		*p*
		*Lower*	*Upper*			*Lower*	*Upper*			*Lower*	*Upper*	
**Sex** (Female vs Male)	2.099	1.312	3.361	**0.002**	1.813	1.101	2.984	**0.019**	1.754	0.975	3.154	0.061
**Education level** (Ref: low)	1.150	0.750	1.763	0.521	0.833	0.517	1.340	0.450	0.978	0.553	1.729	0.939
**Addictiveness: less vs equally**	1.167	0.608	2.240	0.642	2.209	0.961	5.077	0.062	0.980	0.415	2.311	0.963
**Addictiveness: more vs equally**	1.174	0.529	2.604	0.693	2.784	1.064	7.284	**0.037**	1.506	0.551	4.115	0.425
**Harm to self: less vs equally**	0.296	0.163	0.539	**<0.001**	0.455	0.245	0.847	**0.013**	0.504	0.250	1.018	0.056
**Harm to self: more vs equally**	0.177	0.078	0.399	**<0.001**	0.503	0.217	1.168	0.110	0.430	0.158	1.168	0.098
**Harm to others: less vs equally**	0.842	0.333	2.126	0.716	1.290	0.482	3.457	0.612	0.726	0.255	2.066	0.549
**Harm to others: more vs equally**	0.993	0.337	2.927	0.990	0.937	0.289	3.040	0.914	0.805	0.228	2.836	0.735
**Acceptability: less vs equally**	0.975	0.545	1.747	0.933	0.659	0.358	1.213	0.180	0.764	0.367	1.592	0.473
**Acceptability: more vs equally**	1.158	0.479	2.798	0.745	0.505	0.187	1.366	0.179	0.648	0.209	2.013	0.453

AOR: adjusted odds ratio. The logistic regression model includes 384 participants. A total of 16 cases (4%) were excluded from the analysis due to missing values in variables. Missingness primarily resulted from participants selecting ‘prefer not to answer’ on education, or from incomplete responses to the perception items used to calculate 3-category variables.

Participants perceiving flavored products as more addictive were more likely to report past-month e-cigarette use (AOR=2.78; 95% CI: 1.06–7.28, p=0.037). In addition, female participants were more likely than males to report past-month cigarette (AOR=2.10; 95% CI: 1.31–3.36, p=0.002) and e-cigarette use (AOR=1.81; 95% CI: 1.10–2.98, p=0.019). No significant associations were found between perceptions of harm to others or social acceptability and past-month use of any product, or between education level and past-month product use in any models.

Multivariable logistic regression showed no statistically significant associations between participants’ perceptions of flavored products and their past-month use of flavored cigarettes, e-cigarettes, or HTPs (Table 4). Although some odds ratios indicated potential trends, none reached statistical significance.

**Table 4:** Multiple logistic regression of predictors of flavored product use among participants reporting past-month users (Set B), Georgia, April–June 2024 (N=199)

*Predictors*	*Cigarettes*				*E-cigarettes*				*HTPs*			
	*AOR*	*95% CI*		*p*	*AOR*	*95% CI*		*p*	*AOR*	*95% CI*		*p*
		*Lower*	*Upper*			*Lower*	*Upper*			*Lower*	*Upper*	
**Sex** (Female vs Male)	0.770	0.342	1.733	0.527	0.045	0.003	0.612	0.020	0.900	0.279	2.905	0.860
**Education level** (Ref: low)	0.581	0.261	1.292	0.183	2.777	0.422	18.283	0.288	0.634	0.198	2.023	0.441
**Addictiveness: less vs equally**	2.128	0.537	8.432	0.282	1.699	0.102	28.410	0.712	2.570	0.386	17.130	0.329
**Addictiveness: more vs equally**	1.521	0.320	7.226	0.598	0.518	0.023	11.679	0.679	2.668	0.269	26.463	0.402
**Harm to self: less vs equally**	1.264	0.512	3.121	0.612	0.878	0.124	6.196	0.896	1.438	0.383	5.408	0.591
**Harm to self: more vs equally**	0.428	0.074	2.486	0.344	*	*	*	*	3.280	0.471	22.857	0.230
**Harm to others: less vs equally**	0.535	0.147	1.955	0.344	0.218	0.009	5.147	0.345	1.693	0.191	15.016	0.636
**Harm to others: more versus equally**	1.130	0.210	6.091	0.887	*	*	*	*	3.825	0.331	44.187	0.283
**Acceptability: less vs equally**	0.505	0.184	1.391	0.186	1.119	0.086	14.559	0.932	0.314	0.072	1.375	0.124
**Acceptability: more vs equally**	0.776	0.176	3.414	0.737	0.049	0.001	1.666	0.094	0.880	0.084	9.252	0.915

AOR: adjusted odds ratio.

* Unstable estimates produced by SPSS due to small subgroup sizes or limited variability in certain categories.

Multivariable logistic regression examining perceptions in relation to next-year intention to use any flavored tobacco products among non-users of any tobacco products (Table 5) indicated that participants who perceived flavored products as more harmful to others were less likely to report high intention to use flavored cigarettes (AOR=0.040; 95% CI: 0.003–0.622, p=0.022). Similarly, those who perceived flavored products as less harmful to others had lower odds of future use intention of flavored cigarettes (AOR=0.079; 95% CI: 0.012–0.508, p=0.008).

**Table 5:** Multiple logistic regression of predictors of higher intention to use flavored products among non-users (Set C), Georgia, April–June 2024 (N=201)

*Predictors*	*Cigarettes*				*E-cigarettes*				*HTPs*			
	*AOR*	*95% CI*		*p*	*AOR*	*95% CI*		*p*	*AOR*	*95% CI*		*p*
		*Lower*	*Upper*			*Lower*	*Upper*			*Lower*	*Upper*	
**Sex** (Female vs Male)	0.488	0.086	2.758	0.417	0.644	0.294	1.412	0.272	1.133	0.275	4.673	0.863
**Education level** (Ref: low)	0.657	0.192	2.245	0.503	1.017	0.542	1.908	0.959	0.458	0.113	1.863	0.275
**Addictiveness: less vs equally**	0.472	0.105	2.119	0.327	0.716	0.310	1.657	0.435	1.383	0.151	12.654	0.774
**Addictiveness: more vs equally**	0.418	0.054	3.266	0.406	0.777	0.257	2.350	0.655	2.569	0.236	27.945	0.438
**Harm to self: less vs equally**	1.300	0.169	9.997	0.801	0.525	0.218	1.266	0.151	0.348	0.084	1.439	0.145
**Harm to self: more vs equally**	1.800	0.190	17.035	0.608	0.580	0.177	1.901	0.368	0.185	0.016	2.171	0.179
**Harm to others: less vs equally**	0.079	0.012	0.508	**0.008**	2.245	0.446	11.290	0.327	1.180	0.115	12.126	0.889
**Harm to others: more vs equally**	0.040	0.003	0.622	**0.022**	1.279	0.198	8.268	0.796	2.074	0.134	32.111	0.602
**Acceptability: less vs equally**	0.430	0.113	1.636	0.216	0.788	0.338	1.840	0.583	1.604	0.185	13.930	0.669
**Acceptability: more vs equally**	0.698	0.065	7.453	0.766	0.545	0.144	2.056	0.370	2.808	0.231	34.130	0.418

AOR: adjusted odds ratio.

## DISCUSSION

This study found that the majority of participants, aged 18–25 years and Georgian, perceived flavored and unflavored products as equally harmful, addictive, and acceptable. While some studies showed similar perceived harm for flavored and unflavored products^11^, most research has generally shown that youth and young adults perceive flavored tobacco products as less harmful, more appealing, and better tasting than non-flavored tobacco products^8,12,13^. Thus, these findings warrant replication as they may suggest important differences in countries with high tobacco use rates and may be crucial to inform policies specific to flavored tobacco products, particularly products like e-cigarettes that may have implications for harm reduction.

However, an important proportion in the current sample still perceived flavored products more favorably in specific ways, and the difference was especially noticeable in relation to e-cigarettes. In particular, flavored e-cigarettes were more frequently perceived as more socially acceptable, more addictive, and less harmful to self than flavored cigarettes and HTPs. This is not surprising, as flavors have been a primary attraction for e-cigarette users since the products first appeared. Numerous studies, including those from the early years of e-cigarette research, have revealed that flavors significantly increase product appeal among youth and young adults^14-17^. Thus, although according to our study, flavoring is not seen by many Georgian young adults as a key differentiator in general, flavored e-cigarettes are an exception. Their distinct perception profile indicates that young adults may assess flavored e-cigarettes risks differently and more favorably than cigarettes and HTPs. These may be caused by current gaps in Georgia’s tobacco control legislation and enforcement – e-cigarettes are subject to less strict packaging and health warning requirements, and nicotine-free e-cigarettes are not currently regulated at all^9-10^. This regulatory gap makes it difficult to address advertising and flavor-driven promotion under the pretext that such products are nicotine-free. Most importantly, there is a complete absence of regulation on flavorings, allowing flavored products to be widely accessible and appealing to young users.

Perceived harm of flavored products, both to oneself and to others, was found to be important in shaping tobacco use and intentions. A number of studies support the view that perceived risk plays a role in shaping behavior of tobacco using among young adults^12,18^. In this study, participants who believed that flavored products were more harmful to themselves were significantly less likely to report using cigarettes or e-cigarettes in the past month. Among non-users, those who believed flavored cigarettes were more harmful to others were significantly less likely to intend to use them. Interestingly, lower intention was also observed among those who perceived these products as less harmful to others. This can seem paradoxical, but perhaps it is because these individuals simply did not have much interest in smoking tobacco, or perhaps there were other determinants that the current study did not assess. These results suggest that prevention messages should highlight both the personal health risks and harm to others associated with flavored tobacco use, although further research is needed to strengthen this evidence.

Similar to study conducted in US-based study^19^, perceived social acceptability of flavored products was not significantly associated with either past-month use or intention to use in the current study. While perceived addictiveness did not predict use intention, it was significantly associated with past-month e-cigarette use. In particular, participants who viewed e-cigarettes as more addictive were more likely to have used them in the past month. This association between greater perceived addictiveness and past-month use may seem counter-intuitive, as this finding contrasts with previous studies which found that, compared to those who do not use e-cigarettes, those reporting past-month use were less likely to perceive e-cigarettes as harmful a person’s overall health^20^, and perceive e-cigarettes as less addictive^21^. A reason for this result could be that people who use e-cigarettes might realize how addictive they are only after they start using them. It is also possible that just knowing that a product is addictive may not deter young adults from using it, especially if it is flavored or seen as trendy. So, these findings suggest that while addiction warnings are important, they may need to be combined with broader strategies to prevent people from using these products.

### Limitations

Several limitations of the study should be considered. The cross-sectional design limits the ability to conclude causality in tobacco use behaviors and perceptions. Reverse causality is also possible, individuals’ perceptions of harm or addictiveness may be influenced by their product use, rather than the other way around. Self-reported data may be subject to social desirability bias and thus affect the accuracy of responses. This could result in misclassification of product use, perceptions, or other variables, potentially biasing the observed associations. The survey was shared on social media, so participants were not randomly selected. This might have introduced selection bias, meaning the sample may not fully reflect all Georgian young adults. Even though the age range was narrow, other factors we did not measure could still affect the results, a possibility known as residual confounding. These issues may limit how much we can generalize the findings to all youth in Georgia.

## CONCLUSIONS

Georgian young adults in this study perceived flavored products, especially flavored e-cigarettes, as more socially acceptable, more addictive, and less harmful to self than other tobacco products. These findings suggest that stronger public communication and regulatory measures may be warranted to address such perceptions, as the absence of comprehensive anti-tobacco messaging and regulation of flavorings and nicotine-free e-cigarettes could contribute to these trends. While this study highlights important associations between perceptions and tobacco use behaviors or intentions, its cross-sectional design limits causal conclusions. Further research, including prospective cohort studies, is needed to better understand how perceptions influence use over time.

## Supplementary Material



## Data Availability

The data supporting this research are available from the authors on reasonable request.
