# Erratum: Perceptions and use intentions of flavored versus unflavored tobacco products among young adults in Georgia: A cross-sectional study

**DOI:** 10.18332/tpc/215502

**Published:** 2025-12-12

**Authors:** European Publishing Production Team

**Affiliations:** 1 European Publishing Production Team, Heraklion, Greece

**Keywords:** young adults, e-cigarettes, harm perception, addictiveness, flavored tobacco products, social acceptability

**Erratum on:**

**Perceptions and use intentions of flavored versus unflavored tobacco products among young adults in Georgia: A cross-sectional study**

**By Tamar Abuladze, Carla Berg, George Bakhturidze, Lela Sturua**

**Tobacco Prevention & Cessation, Volume 11, Issue October, Pages 1–11**

**Publish date: 6 October 2025**

DOI: https://doi.org/10.18332/tpc/208691

In the original article, in the eleventh page, the **Disclaimer** section was missing.

It has been now corrected as follows:

## DISCLAIMER

G. Bakhturidze, Editorial Board member of the journal, had no involvement in the peer-review or acceptance of this article and had no access to information regarding its peer-review. Full responsibility for the editorial process for this article was delegated to a handling editor of the journal.

The mentioned changes are corrected also online.

