# Supplementary material for: Perceptions and use intentions of flavored versus unflavored tobacco products among young adults in Georgia: A cross-sectional study
Source: Tob Prev Cessat. 2025 Oct 6;11:10.18332/tpc/208691. doi: 10.18332/tpc/208691 (PMC12498478; doi:10.18332/tpc/208691)
Supplement: Supplementary file 1 [file TPC-11-46-s1.pdf]

Supplementary file

Supplementary Table 1a. Participant characteristics and bivariate associations with whether participant perceives flavored products more addictive, harmful (to self and to others), and acceptable compared to unflavored products (N=400), Georgia, April-June, 2024 (Cross-sectional Survey)									
	**Total sample N=400 (100%)**	**Addictive**				**Harmful to self**			
		**Less N=63 (15.8%)**	**Equally N=284 (71.0%)**	**More N=53 (13.3%)**		**Less N=59 (14.8%)**	**Equally N=271 (67.8%)**	**More N=70 (17.5%)**	
**Variables**	**N (%)**	**N (%)**	**N (%)**	**N (%)**	**p-value**	**N (%)**	**N (%)**	**N (%)**	**p-value**
Sociodemographics									
**Sex, N (%)**					0.828				0.330
Male	108 (27.0%)	19 (30.2%)	75 (26.4%)	14 (26.4%)		19 (32.2%)	67 (24.7%)	22 (31.4%)	
Female	292 (73.0%)	44 (69.8%)	209 (73.6%)	39 (73.6%)		40 (67.8%)	204 (75.3%)	48 (68.6%)	

**Education level, N (%)**	(Valid N=394)				0.113				0.944
No higher education	179 (45.4%)	26 (41.3%)	122 (43.9%)	31 (58.5%)		28 (47.5%)	120 (45.1%)	31 (44.9%)	
Higher education	215 (54.6%)	37 (58.7%)	156 (56.1%)	22 (41.5%)		31 (52.5%)	146 (54.9%)	38 (55.1%)	
**Employment status, N (%)**	(Valid N=387)				0.155				0.235
Employed	136 (35.1%)	29 (46.0%)	94 (34.6%)	13 (25.0%)		15 (26.8%)	99 (37.4%)	22 (33.3%)	
Student	217 (56.1%)	29 (46.0%)	156 (57.4%)	32 (61.5%)		33 (58.9%)	143 (54.0%)	41 (62.1%)	
Unemployed	34 (8.8%)	5 (7.9%)	22 (8.1%)	7 (13.5%)		8 (14.3%)	23 (8.7%)	3(4.5%)	
**Annual income, N (%)**					0.511				0.622
<15000 GEL	308 (77.0%)	47 (74.6%)	217 (76.4%)	44 (83.0%)		47 (79.7%)	210 (77.5%)	51 (72.9%)	
>15 000 Gel	92 (23.0%)	16 (25.4%)	67 (23.6%)	9 (17.0%)		12 (20.3%)	61 (22.5%)	19 (27.1%)	

Note: Percentages in parentheses represent column percentages, showing the distribution of sociodemographic and user status groups within each perception category (e.g., "more harmful," etc.).

**Supplementary Table 1b. Participant characteristics and bivariate associations with whether participant perceives flavored products more addictive, harmful (to self and to others), and acceptable compared to unflavored products (N=400), Georgia, April-June, 2024 (Cross-sectional Survey)**									
		**Perceive flavored products as more…**							
	**Total sample N=400 (100%)**	**Harmful to others**				**Acceptable**			
		**Less N=54 (13.5%)**	**Equally N=321 (80.3%)**	**More N=25 (6.25%)**		**Less N=39 (9.8%)**	**Equally N=293 (73.3%)**	**More N=68 (17.0%)**	
**Variables**	**N (%)**	**N (%)**	**N (%)**	**N (%)**	**p-value**	**N (%)**	**N (%)**	**N (%)**	**p-value**
Sociodemographics									
**Sex, N (%)**					0.319				0.777
Male	108 (27.0%)	14 (25.9%)	84 (26.2%)	10 (40.0%)		9 (23.1%)	79 (27.0%)	20 (29.4%)	
Female	292 (73.0%)	40 (74.1%)	237 (73.8%)	15 (60.0%)		30 (76.9%)	214 (73.0%)	48 (70.6%)	

**Education level, N (%)**	(Valid N=394)				0.247				0.299
No higher education	179 (45.4%)	26 (48.1%)	146 (46.2%)	7 (29.2%)		20 (52.6%)	125 (43.1%)	34 (51.5%)	
Higher education	215 (54.6%)	28 (51.9%)	170 (53.8%)	17 (70.8%)		18 (47.4%)	165 (56.9%	32 (48.5%)	
**Employment status, N (%)**	(Valid N=387)				0.564				0.084
Employed	136 (35.1%)	17 (32.1%)	110 (35.4%)	9 (39.1%)		9 (23.7%)	105 (36.7%)	22 (34.9%)	
Student	217 (56.1%)	32 (60.4%)	171 (55.0%)	14 (60.9%)		27 (71.1%)	159 (55.6%)	31 (49.2%)	
Unemployed	34 (8.8%)	4 (7.5%)	30 (9.6%)	0 (0%)		2 (5.3%)	22 (7.7%)	10 (15.9%)	
**Annual income, N (%)**					0.493				0.812
<15000 GEL	308 (77%)	45 (83.3%)	244 (76.0%)	19 (76%)		29 (74.4%)	228 (77.8%)	51 (75%)	
>15 000 Gel	92 (23%)	9 (16.7%)	77 (24.0%)	6 (24%)		10 (25.6%)	65 (22.2%)	17 (25%)	

Note: Percentages in parentheses represent column percentages, showing the distribution of sociodemographic and user status groups within each perception category (e.g., "more harmful," etc.).

Supplementary Table 2a. Bivariate associations with whether participant perceives flavored products more addictive, harmful (to self and to others), and acceptable compared to unflavored products (N=400), Georgia, April-June, 2024 (Cross-sectional Survey)

	**Total sample N=400 (100%)**	**Addictive**				**Harmful to self**			
		**Less N=63 (15.8%)**	**Equally N=284 (71.0%)**	**More N=53 (13.3%)**		**Less N=59 (14.8%)**	**Equally N=271 (67.8%)**	**More N=70 (17.5%)**	
**Variables**	**N (%)**	**N (%)**	**N (%)**	**N (%)**	**p-value**	**N (%)**	**N (%)**	**N (%)**	**p-value**
***Past-month use,* N (%)**									
Cigarettes									
No	234 (58.5%)	36 (57.1%)	167 (58.8%)	31 (58.5%)	0.971	42 (71.2%)	170 (62.7%)	22 (31.4%)	**<0.001**

Yes	166 (41.5%)	27 (42.9%)	117 (41.2%)	22 (41.5%)		17 (28.8%)	101 (37.3%)	48 (68.6%)	
Unflavored	128 (32.0%)	21 (33.3%)	88 (31%)	19 (35.8%)	0.849	15 (25.4%)	76 (28.0%)	37 (52.9%)	**<0.001**
Flavored	38 (9.5%)	6 (9.5%)	29 (10.2%)	3 (5.7%)		2 (3.4%)	25 (9.2%)	11 (15.7%)	
E-cigarettes									
No	300 (75.0%)	44 (69.8%)	211 (74.3%)	45 (84.9%)	0.154	45 (76.3%)	210 (77.5%)	45 (64.3%)	0.073
Yes	100 (25.0%)	19 (30.2%)	73 (25.7%)	8 (15.1%)		14 (23.7%)	61 (22.5%)	25 (35.7%)	
Unflavored	8 (2.0%)	2 (3.2%)	5 (1.8%)	1 (1.9%)	0.387	0 (0%)	5 (1.8%)	3 (4.3%)	0.182
Flavored	91 (22.8)	17 (27.0%)	67 (23.6%)	7 (13.2%)		14 (23.7%)	56 (20.7%)	21 (30.0%)	
HTPs									
No	340 (85%)	50 (79.4%)	245 (86.3%)	45 (84.9%)	0.382	51 (86.4%)	236 (87.1%)	53 (75.7%)	0.056
Yes	60 (15%)	13 (20.6%)	39 (13.7%)	8 (15.1%)		8 (13.6%)	35 (12.9%)	17 (24.3%)	
Unflavored	32 (8.0%)	6 (9.5%)	20 (7.0%)	6 (11.3%)	0.449	3 (5.1%)	19 (7%)	10 (14.3%)	0.092
Flavored	29 (7.2%)	7 (11.1%)	20 (7.0%)	2 (3.8%)		5 (8.5%)	16 (5.9%)	8 (11.4%)	

Any product									
No	201(50.2%)	26 (41.3%)	146 (51.4%)	29 (54.7%)	0.271	35 (59.3%)	149 (55.0%)	17 (24.3%)	**<0.001**
Yes	199 (49.8%)	37 (58.7%)	138 (48.6%)	24 (45.3%)		24 (40.7%)	122 (45.0%)	53 (75.7%)	
Use intentions	**M (SD)**	**M (SD)**	**M (SD)**	**M (SD)**	**p-value**	**M (SD)**	**M (SD)**	**M (SD)**	**p-value**
Unflavored cigarettes	2.60 (2.18)	3.02 (2.35)	2.49 (2.10)	2.72 (2.35)	0.205	2.47 (2.19)	2.42 (2.10)	3.41 (2.31)	**0.003**
Flavored cigarettes	1.93 (1.69)	2.03 (1.86)	1.93 (1.66)	1.81 (1.63)	0.783	2.20 (1.90)	1.85 (1.65)	2.00 (1.62)	0.326
Unflavored e-cigarettes	1.54 (1.37)	1.76 (1.64)	1.50 (1.31)	1.43 (1.28)	0.337	1.59 (1.48)	1.56 (1.42)	1.40 (1.00)	0.650
Flavored e-cigarettes	2.16 (1.75)	2.33 (1.78)	2.07 (1.72)	2.40 (1.89)	0.323	2.44 (1.98)	2.14 (1.79)	2.00 (1.38)	0.343
Unflavored HTPs	1.61 (1.49)	1.73 (1.58)	1.60 (1.50)	1.51 (1.33)	0.717	1.64 (1.49)	1.58 (1.51)	1.67 (1.43)	0.888
Flavored HTPs	1.57 (1.42)	1.70 (1.57)	1.57 (1.43)	1.43 (1.14)	0.605	1.78 (1.61)	1.56 (1.45)	1.44 (1.09)	0.392

Note: Percentages in parentheses represent column percentages, showing the distribution of sociodemographic and user status groups within each perception category (e.g., “more harmful,” etc.).

Supplementary Table 2b. Bivariate associations with whether participant perceives flavored products more addictive, harmful (to self and to others), and acceptable compared to unflavored products (N=400), Georgia, April-June, 2024 (Cross-sectional Survey)

		**Perceive flavored products as more…**							
	**Total sample N=400 (100%)**	**Harmful to others**				**Acceptable**			
		**Less N=54 (13.5%)**	**Equally N=321 (80.3%)**	**More N=25 (6.25%)**		**Less N=39 (9.8%)**	**Equally N=293 (73.3%)**	**More N=68 (17.0%)**	
**Variables**	**N (%)**	**N (%)**	**N (%)**	**N (%)**	**p-value**	**N (%)**	**N (%)**	**N (%)**	**p-value**
***Past-month use*, N (%)**									
Cigarettes									
No	234 (58.5%)	32 (59.3%)	192 (59.8%)	10 (40%)	0.152	20 (51.3%)	174 (59.4%)	40 (58.8%)	0.627
Yes	166 (41.5%)	22 (40.7%)	129 (40.2%)	15 (60%)		19 (48.7%)	119 (40.6%)	28 (41.2%)	
Unflavored	128 (32%)	16 (29.6%)	102 (31.8%)	10 (40%)	0.230	14 (35.9%)	94 (32.1%)	20 (29.4%)	0.768

Flavored	38 (9.5%)	6 (11.1%)	27 (8.4%)	5 (20.0%)		5 (12.8%)	25 (8.5%)	8 (11.8%)	
E-cigarettes									
No	300 (75%)	42 (77.8%)	240 (74.8%)	18 (72.0%)	0.839	31 (79.5%)	222 (75.8%)	47 (69.1%)	0.414
Yes	100 (25%)	12 (22.2%)	81 (25.2%)	7 (28.0%)		8 (20.5%)	71 (24.2%)	21 (30.9%)	
Unflavored	8 (2%)	0 (0%)	7 (2.2%)	1 (4%)	0.774	2 (5.1%)	5 (1.7%)	1 (1.5%)	0.299
Flavored	91 (22.8)	12 (22.2%)	74 (23.1%)	5 (20.0%)		6 (15.4%)	65 (22.2%)	20 (29.4%)	
HTPs									
No	340 (85%)	45 (83.3%)	276 (86.0%)	19 (76.0%)	0.378	33 (84.6%)	251 (85.7%)	56 (82.4%)	0.787
Yes	60 (15%)	9 (16.7%)	45 (14.0%)	6 (24.0%)		6 (15.4%)	42 (14.3%)	12 (17.6%)	
Unflavored	32 (8%)	3 (5.6%)	25 (7.8%)	4 (16%)	0.256	3 (7.7%)	25 (8.5%)	4 (5.9%)	0.428
Flavored	29 (7.2)	6 (11.1%)	20 (6.2%)	3 (12.0%)		4 (10.3%)	17 (5.8%)	8 (11.8%)	
Any product									
No	201(50.2%)	30 (55.6%)	165 (51.4%)	6 (24.0%)	**0.022**	20 (51.3%)	150 (51.2%)	31 (45.6%)	0.700

Yes	199 (49.8%)	24 (44.4%)	156 (48.6%)	19 (76.0%)		19 (48.7%)	143 (48.8%)	37 (54.4%)	
**Use intentions**	**M (SD)**	**M (SD)**	**M (SD)**	**M (SD)**	**p-value**	**M (SD)**	**M (SD)**	**M (SD)**	**p-value**
Unflavored cigarettes	2.60 (2.18)	2.26 (1.92)	2.59 (2.21)	3.56 (2.16)	**0.045**	3.18 (2.41)	2.58 (2.15)	2.37 (2.15)	0.169
Flavored cigarettes	1.93 (1.69)	1.72 (1.39)	1.92 (1.69)	2.52 (2.14)	0.143	1.92 (1.77)	1.98 (1.75)	1.74 (1.36)	0.570
Unflavored e-cigarettes	1.54 (1.37)	1.39 (1.14)	1.47 (1.27)	2.72 (2.23)	**<0.001**	1.82 (1.64)	1.54 (1.37)	1.37 (1.17)	0.256
Flavored e-cigarettes	2.16 (1.75)	2.04 (1.77)	2.13 (1.72)	2.72 (2.05)	0.236	2.33 (1.83)	2.14 (1.74)	2.13 (1.80)	0.805
Unflavored HTPs	1.61 (1.49)	1.48 (1.16)	1.55 (1.44)	2.56 (2.24)	**0.004**	1.64 (1.41)	1.66 (1.57)	1.35 (1.12)	0.301
Flavored HTPs	1.57 (1.42)	1.50 (1.16)	1.52 (1.37)	2.36 (2.20)	**0.015**	1.59 (1.53)	1.58 (1.41)	1.51 (1.40)	0.939

Note: Percentages in parentheses represent column percentages, showing the distribution of sociodemographic and user status groups within each perception category (e.g., "more harmful," etc.).

© 2025 Abuladze T. et al.
